# Expression stromale de CD10 dans les cancers du sein: marqueur de mauvais pronostic

**DOI:** 10.11604/pamj.2020.37.70.20223

**Published:** 2020-09-17

**Authors:** Dhouha Bacha, Anissa Ben Amor, Farah Ben Farhat, Sana Ben Slama, Ahlem Lahmar, Saadia Bouraoui, Amel Triki

**Affiliations:** 1 Service d’Anatomie Pathologique, Hôpital Mongi Slim, La Marsa, Tunisie,; 2 Service de Gynécologie Obstétrique, Hôpital Mongi Slim, La Marsa, Tunisie

**Keywords:** Tumeurs du sein, CD10, cellules stromales, pronostic, Breast cancer, CD10, stromal cells, prognostic

## Abstract

**Introduction:**

les marqueurs du stroma carcinomateux comme la classe de différenciation 10 (CD10) seraient corrélés à un potentiel invasif et métastatique de plusieurs types de cancers en favorisant la croissance tumorale et l’apparition de métastases. Pour les carcinomes mammaires, sa valeur pronostique reste encore controversée avec des résultats discordants. Les objectifs de notre étude étaient d’étudier l’expression stromale de CD10 dans les carcinomes mammaires et d’évaluer la valeur pronostique de cette expression.

**Méthodes:**

notre étude était rétrospective, descriptive et pronostique. Elle avait intéressé 57 patientes porteuses de carcinomes invasifs de type non spécifique, colligés au service d’anatomie pathologique de l’hôpital Mongi Slim, sur une période de 38 mois. L’étude de CD10 a été réalisée par immunohistochimie et son interprétation était basée sur un score semi-quantitatif en trois catégories avec des seuils de 10 et 30%.

**Résultats:**

l’âge moyen de nos patientes était de 56,4 ans. Vingt-huit patientes (49%) avaient un marquage stromal de CD10, la moitié était de score 1 (faible) et l’autre moitié de score 2 (fort). Ce marquage diminuait significativement la survie sans récidives (p=0,001). Il n’y a en revanche pas d’influence sur la survie globale (p=0,84). L’étude de corrélation avait montré que l’expression stromale de CD10 était significativement corrélée à 12 facteurs de mauvais pronostic dans les cancers du sein.

**Conclusion:**

l’expression de CD10 dans le stroma des carcinomes mammaires invasifs constitue un facteur de mauvais pronostic, prédictif d’une faible survie sans récidives et est associée à un potentiel invasif et métastatique élevé.

## Abstract (en)

**Introduction:**

markers in breast cancer stem cells, such as cluster of differentiation 10 (CD10), would be correlated with invasive and metastatic potential of several types of cancer, contributing to tumor growth and metastases. In patients with breast cancer, its prognostic value is still controversial, given the discrepancy of results. The purpose of the study was to study CD10 expression in stromal cells of patients with breast cancer as well as to evaluate the prognostic value of this expression.

**Method:**

we conducted a retrospective, descriptive and prognostic study. It involved 57 patients with invasive cancer of no special type, whose data were collected in the Department of Pathological Anatomy at the Mongi Slim Hospital over a 38-month period. CD10 expression was studied immunohistochemically and interpreted by semi-quantitative scoring system based on three categories with thresholds of 10 and 30%.

**Results:**

the average age of patients was 56.4 years. Twenty-eight patients (49%) had labeled CD10 stromal, half of patients had a score of 1 (low) and the other half had 2 (high). This labeling significantly reduced recurrence-free survival (p=0.001). However, it had no influence on overall survival (p=0.84). The correlation study showed that CD10 expression in stromal cells was significantly correlated with 12 poor prognostic factors in patients with breast cancer.

**Conclusion:**

CD10 expression in stromal cells of invasive breast cancer is a poor prognostic factor, predictive of poor survival without recurrence and associated with a high invasive and metastatic potential.

## Introduction

Le cancer du sein est le 2^e^cancer le plus fréquent au monde et le premier cancer de la femme, précédant le cancer colorectal et celui du poumon [1]. Il représente la cause la plus fréquente de décès par cancer chez les femmes dans le monde (522 000 décès en 2012) [2]. De nombreux facteurs histo-pronostiques et phénotypiques sont utilisés pour évaluer le potentiel évolutif des cancers du sein, comme le type histologique, le grade tumoral, la classification pTNM, l’index de prolifération, les récepteurs hormonaux et le statut “Human Epidermal Growth Factor Receptor 2 (HER 2)”. L’étude de ces facteurs et les progrès thérapeutiques réalisés ces dernières décennies avaient contribué à améliorer la prise en charge des cancers du sein pour devenir de plus en plus personnalisée. Toutefois, son pronostic demeure encore grave du fait de la fréquence des récidives locales et des métastases, qui sont responsables d’un grand nombre d’échecs thérapeutiques [3]. De ce fait, l’étude de nouveaux marqueurs prédictifs du potentiel invasif et métastatique des cancers du sein est primordiale.

Parmi ces marqueurs, ceux du stroma carcinomateux et en particulier les macro-molécules de la matrice extra-cellulaire (MEC), acquièrent de plus en plus un intérêt pronostique dans l’étude de plusieurs cancers. Ils sont en passe de devenir le nouveau défi des pathologistes. Parmi ces molécules de la MEC, la classe de différenciation 10 (CD10) est une métalloprotéinase produite par les myofibroblastes du stroma carcinomateux. L’expression stromale de ce marqueur serait corrélée à un potentiel agressif de plusieurs types de cancers comme le cancer colorectal, pulmonaire, de la prostate, du rein et du foie, en favorisant la croissance tumorale et l’apparition de métastases [4-9]. Pour les carcinomes mammaires, la valeur pronostique de l’expression stromale de CD10 reste encore controversée avec des résultats discordants. Les objectifs de notre étude étaient: d’étudier par immunohistochimie (IHC) l’expression stromale de CD10 dans les carcinomes mammaires et d’évaluer la valeur pronostique de cette expression.

## Méthodes

Notre étude était rétrospective, descriptive et pronostique.

**Patientes:** l’étude avait inclus 57 patientes non consécutives, porteuses de carcinomes invasifs de type non spécifique (NST) colligés au service d’anatomie pathologique de l’hôpital Mongi Slim, sur une période de 38 mois (janvier 2014 - mars 2017). Les patientes ayant eu un traitement néo-adjuvant par chimiothérapie (CT) n’ont pas été incluses dans cette étude. L’anonymat des patients a été respecté.

**Recueil des données:** le recueil des données cliniques était basé sur les dossiers des patientes. Les informations recueillies portaient sur l’âge de la patiente, ses antécédents personnels et familiaux, le motif de sa consultation au service de gynécologie obstétrique et les données de l’examen physique, biologique et radiologique. Le geste chirurgical réalisé et les données évolutives ont été également relevés. Les patientes perdues de vue ont été contactées par téléphone. Pour les données radiologiques, leur interprétation s’était basée sur la classification ACR de BIRADS [10]. Le recueil des données anatomo-pathologiques était basé sur les comptes rendus pathologiques pour l’examen macroscopique. Pour l’examen histologique, il était basé sur la relecture des lames des biopsies ou des pièces d’exérèse chirurgicale. Pour les micro-biopsies, la taille du (ou des) cylindre(s) biopsique(s) a été relevée. Pour les pièces opératoires, la nature de la pièce de résection tumorale et celle des autres prélèvements adressés ont été notées. La topographie, l’aspect et la taille de la tumeur ont été relevés, ainsi que les limites de résection chirurgicales. La taille de la tumeur était celle trouvée à l’imagerie pour les patientes ayant eu une micro-biopsie mammaire et celle relevée sur la pièce opératoire pour les patientes ayant eu une résection chirurgicale.

**Etude anatomo-pathologique:** l’expression de CD10 était évaluée par une étude immuno-histochimique (IHC). Tous les blocs choisis comportaient un témoin interne fortement positif, représenté par les cellules myoépithéliales des lobules mammaires péri-tumoraux, pour la validation de la technique. Une relecture des lames colorées à l’Hématéine Eosine (HE) a été effectuée avant l’étude IHC, dans l’objectif de choisir un bloc représentatif et adéquat de la tumeur pour une interprétation optimale de l’immuno-marquage. Nous avons choisi les blocs où le contingent infiltrant de la tumeur était étendu, non nécrosé et non écrasé. Pour l’étude de corrélation pronostique entre l’expression stromale de CD10 et les paramètres clinico-pathologiques, nous avons relevé les variables suivantes: l’âge ≤40 ans, le type inflammatoire du carcinome, la taille tumorale >20mm, le grade SBR modifié par Ellis et Elston (Grade de Nottingham), la différenciation tumorale, les atypies nucléaires, l’index mitotique, le profil des récepteurs hormonaux (aux œstrogènes et à la progestérone), le statut HER2, l’index de prolifération Ki67, les sous types phénotypiques, les emboles vasculaires, les métastases ganglionnaires, les métastases systémiques au diagnostic et les récidives (locales et systémiques).

L’étude IHC a été réalisée en utilisant l’anticorps CD10 prêt à l’emploi (LEICA, clone 56C6) et l’automate Bond Max de Leica. L’évaluation de l’expression de CD10 a été réalisée par un seul pathologiste (DB). Les cellules comptabilisées étaient celles du stroma tumoral, associant les myofibroblastes, les cellules de la lignée myéloïde et les cellules endothéliales, évaluées dans tout le prélèvement, pour aboutir à une moyenne des cellules marquées. Nous avons utilisé un score semi-quantitatif, testé dans l’étude de Puri, pour évaluer la proportion des cellules stromales positives, indépendamment de l’intensité du marquage [11]. Ce score individualise 3 catégories: *résultat négatif (score 0):* une moyenne de <10% de cellules stromales positives sur l’ensemble des micro-biopsies ou sur le prélèvement choisi; *résultat faible (score 1):* une moyenne de cellules stromales positives entre 10 et 30% sur l’ensemble des micro-biopsies ou sur le prélèvement choisi; *résultat fort (score 2):* une moyenne de >30% de cellules stromales positives sur l’ensemble des micro-biopsies ou sur le prélèvement choisi.

**Analyse statistique:** les informations ont été saisies et analysées par le logiciel Statistical Package for the Social Sciences (SPSS) version 25. Les proportions ont été présentées avec un intervalle de confiance à 95%. Les variables quantitatives continues étaient évaluées par la moyenne et l’écart type et les variables qualitatives par un pourcentage. La comparaison des moyennes a été effectuée en utilisant le test de *student*. La recherche d’une corrélation entre l’expression stromale de CD10 et les paramètres clinico-pathologiques a été effectuée en utilisant le test de Khi-2 de Pearson. En cas d’effectif inférieur à 5, une correction par le test de Fisher a été effectuée. La survie globale (SG) et la survie sans récidives (SSR) étaient déterminées en établissant des courbes de survie selon la méthode de Kaplan-Meier. La date d’origine étant le jour de la chirurgie. La recherche des facteurs pronostiques de survie a été effectuée en analyse univariée (facteur par facteur) en comparant les courbes de survie par le test du Log Rank. Les facteurs étudiés étaient les mêmes que ceux analysés dans l’étude de corrélation, en ajoutant le CD10. Afin d’identifier les facteurs de risque liés de façon indépendante à la SG et la SSR, nous avons conduit une analyse multivariée en régression de Cox, méthode pas à pas descendante. Cette méthode était basée sur l’introduction de tous les facteurs dont les degrés de signification «p» était <0,2 en analyse univariée, en ajoutant l’âge. L’analyse multivariée avait permis de calculer des risques relatifs ajustés, mesurant le rôle propre de chaque facteur sur la SG et la SSR. Dans tous les tests statistiques, le seuil de signification était fixé à 0,05.

## Résultats

**Caractéristiques des patientes (**Tableau 1**):** l’âge moyen de nos patientes au moment du diagnostic était de 56,4 ans avec une médiane de 56 ans et des extrêmes allant de 34 à 83 ans. La découverte d’une masse palpable était la circonstance du diagnostic la plus fréquente, notée chez 42 patientes (74%). Le cancer du sein a été découvert de façon fortuite chez quatre patientes et lors d’un dépistage chez 2 patientes. Neuf patientes présentaient un carcinome de type inflammatoire. Le bilan radiologique avait permis de bien individualiser la tumeur, ses rapports et son bilan d’extension. Une mammographie a été réalisée chez toutes les patientes et a montré une anomalie indéterminée ou suspecte, nécessitant une vérification histologique (ACR4) chez 23 patientes et une anomalie évocatrice de cancer (ACR5) chez 34 patientes (59%). Cinquante et une patientes (96%) avaient bénéficié d’une échographie mammaire et 14 patientes d’une imagerie par résonnance magnétique (IRM) mammaire. Pour le bilan d’extension, une tomodensitométrie thoraco-abdomino-pelvienne (TDM TAP) et une scintigraphie osseuse ont été pratiquées chez respectivement 45 et 30 patientes. Ce bilan avait montré la présence de métastases à distance au moment du diagnostic chez quatre patientes (osseuse et pulmonaire chez une patiente chacune, et hépatique chez deux patientes).

**Tableau 1:** caractéristiques clinico-pathologiques des 57 patientes de notre série

Variables		Effectifs	Pourcentage
**Age**	≤40ans	3	5,3%
	>40ans	54	94,7%
**Taille tumorale**	>20 mm	38	67%
	≤ 20mm	19	33%
**Différenciation tumorale**	Bien différenciée	13	23%
	Moyennement différenciée	10	17,5%
	Peu différencié	34	59,5%
**Grade de Nottingham**	I	20	35%
	II	26	46%
	III	11	19%
**Marquage stromal de CD10**	Score 0	29	51%
	Score 1	14	24,5%
	Score 2	14	24,5%
**Récepteurs aux œstrogènes**	Positifs	41	72%
	Négatifs	16	28%
**Récepteurs à la progestérone**	Positifs	28	49%
	Négatifs	29	51%
**Profil HER2**	Amplifié	17	30%
	Non amplifié	40	70%
**Ki67**	≥14%	28	49%
	<14%	29	41%
**Profil phénotypique**	Luminal A	26	46%
	Luminal B	14	24,5%
	Triple négatif	9	16%
	HER2+	8	13,5%
**Présence de métastases au diagnostic**	Ganglionnaires	19	33,5%
	Systémiques	4	7%
**Survenue de récidives**	Locales	16	28%
	Systémique	7	12,3%
**Décès**		13	22,8%

**Figure 1:**
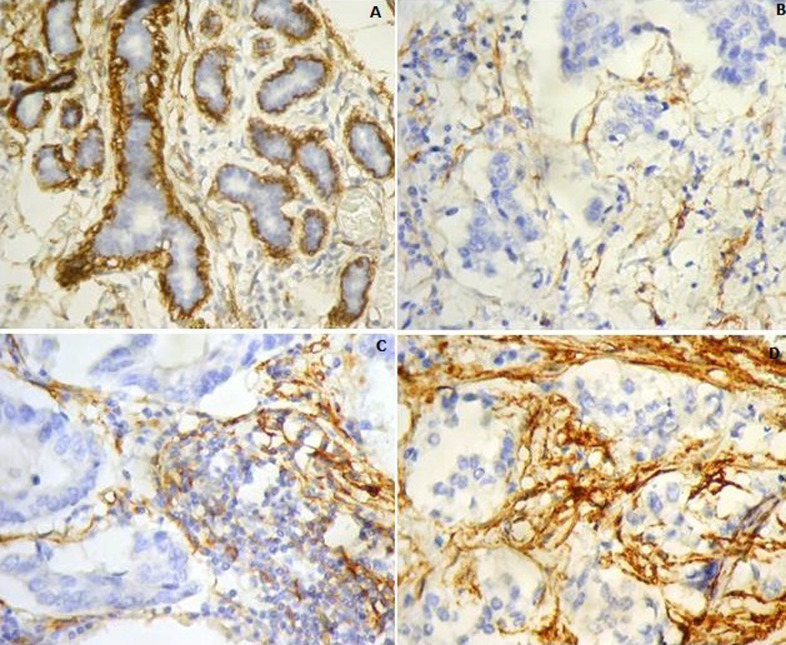
A) expression du CD10 dans les carcinomes mammaires (CD4x200); témoin interne: marquage des cellules myoépithéliales des acini du parenchyme mammaire normal; B) score 0: marquage de moins de 10% des cellules stromales; C) score 1: marquage de 25% des cellules stromales; D) score 2: expression de 80% des cellules stromales

**Etude anatomo-pathologique:** l’étude avait intéressé 30 micro-biopsies mammaires (53%), 20 mastectomies avec curage axillaire et sept tumorectomies avec curage axillaire. Il n’y avait pas de tumeurs bifocales. Ainsi, chaque patiente présentait un seul carcinome infiltrant. Les micro-biopsies avaient une taille globale comprise entre 7mm et 28mm (moyenne de 18mm). La taille tumorale moyenne au diagnostic était de 31,4mm. Quarante patientes (70%) avaient des atypies marquées. Quatorze patientes avaient un nombre de mitoses ≥10/mm^2^. Les carcinomes infiltrants étaient majoritairement de grade II (46%). Les limites de résection sur les pièces opératoires (tumorectomies et mastectomies) étaient saines. Sur les 20 pièces de mastectomie, il n’y avait pas de maladie de Paget du mamelon. Dix-neuf patientes avaient un carcinome in situ (CIS) associé, de bas grade dans tous les cas. Concernant l’étude IHC par le CD10, en dehors du marquage des cellules myoépithéliales des acini et des canaux mammaires normaux, pris comme témoins interne pour la validation de la technique, les cellules endothéliales et les adipocytes étaient dans de rares cas CD10+ (Figure 1 A). Pour les 19 patientes qui avaient un CIS associé, aucune n’avait de marquage des cellules stromales. Nous avons noté un marquage des cellules myoépithéliales des canaux dans tous les cas et un marquage des cellules carcinomateuses chez 11 patientes. En ce qui concerne le carcinome infiltrant, 28 patientes (49%) avaient un marquage stromal pour CD10, dont la moitié de score 1 et l’autre moitié de score 2. De plus, 5 patientes avaient un marquage des cellules carcinomateuses (Figure 1 B, C, D).

**Figure 2:**
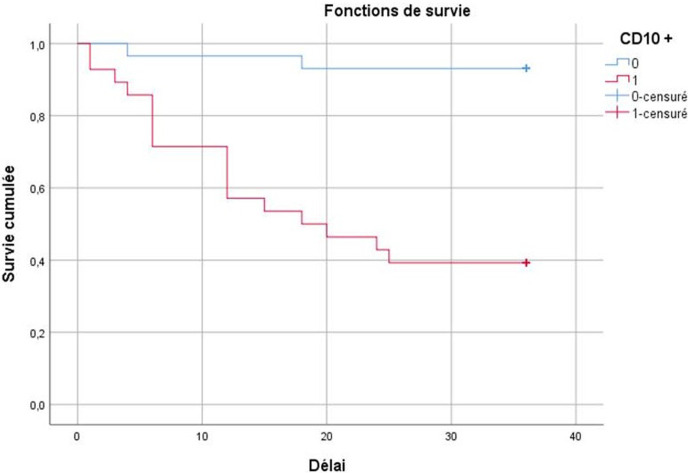
courbes de la survie sans récidives en fonction de l’expression stromale de CD10

**Evolution et survie:** la durée moyenne du suivi des patientes était de 28 mois. Une récidive locale (mammaire) était notée dans 16 cas dans un délai moyen de 11,5 mois. Des récidives systémiques étaient notées dans 7 cas lors du suivi, dans un délai moyen de 8 mois. Il s’agissait de métastases osseuses (n= 2), pulmonaires (n= 2) et hépatiques (n= 3). Treize patientes étaient décédées dans un délai moyen de 16,38 mois. La durée moyenne de SG et SSR, était respectivement de 31,5 mois±1,3 mois et de 27,6 mois±1,7 mois. Le taux moyen de SG était de 77%. Le taux moyen de SSR était de 66%. En étudiant la SG en fonction des facteurs pronostiques, les récidives systémiques diminuaient significativement la SG en analyse univariée (p=0,003). L’analyse multi-variée avait montré que les récidives systémiques étaient un facteur indépendant pour la SG (OR: 4,9; 95% CI: 1,5-16,15; p=0,008). En ce qui concerne la SSR, l’analyse uni-variée avait montré que le type inflammatoire du carcinome, la taille tumorale >20mm, le grade III, le profil «triple négatif», le Ki67≥14%, le statut HER2 amplifié la présence d’emboles vasculaires, les métastases ganglionnaires, le marquage stromal de CD10 (Figure 1, Figure 2) et la présence de métastases systémiques au diagnostic diminuaient significativement la SSR. L’analyse multi-variée avait montré que l’expression stromale de CD10 (OR: 3,024; 95% IC: 1,59-5,752; p=0,001) (Figure 1), la présence d’emboles vasculaires (p=0,004, OR: 4,663; 95% IC: 1,637-13,284; p=0,004) et le type inflammatoire du cancer (OR: 4,469; 95% IC: 1.373-14.548; p=0,013) étaient des facteurs pronostiques indépendants pour la SSR.

**Corrélation entre l’expression stromale de CD10 et les autres facteurs pronostiques:** dans notre série, l’expression stromale de CD10 était significativement corrélée à une taille tumorale >20mm, à un grade SBR III, à l’absence de RE, aux profils «luminal A», «HER2» et «triple négatif», au taux de Ki67≥14%, au statut HER2 amplifié, à la présence de métastases systémiques au moment du diagnostic, aux métastases ganglionnaires, et à la présence de récidives locales et systémiques (Tableau 2).

**Tableau 2:** caractéristiques clinico-pathologiques des patientes selon l’expression stromale de CD10

Variables	CD10+	CD10-	p
**Age ≤ 40 ans**	3	0	0,07
**Type inflammatoire du carcinome**	6	3	0,251
**Taille tumorale >20mm**	23	15	**0,015**
**Carcinome peu différencié**	19	15	0,215
**Carcinome de grade III**	10	1	**0,002**
**Atypies nucléaires marquées**	21	19	0,434
**Mitoses ≥10/mm^2^**	10	4	0,055
**RE+**	14	27	**0,001**
**RP+**	12	16	0,352
**HER2 amplifié**	13	4	**0,007**
**Ki67 ≥14%**	23	5	**0,001**
**Luminal A**	4	22	**0,001**
**Luminal B**	9	5	0,113
**Triple négatif**	8	1	**0,009**
**HER2**	7	1	**0,019**
**Métastases systémiques au moment du diagnostic**	4	0	**0,03**
**Emboles vasculaires**	10	5	0,113
**Métastases ganglionnaires**	14	5	**0,009**
**Récidives systémiques**	7	0	**0,004**
**Récidives locales**	14	2	**0,001**

**RE+ :** Récepteurs aux œtrogènes positifs **RP+ :** Récepteurs à la progestérone positifs

## Discussion

Les marqueurs du stroma carcinomateux acquièrent de plus en plus un intérêt pronostique dans l’étude de plusieurs types de cancers. Dans notre étude, nous avons testé l’un de ces marqueurs, le CD10, chez 57 patientes porteuses de carcinomes invasifs NST. Nous avons trouvé une expression positive chez 28 patientes (49%), dont la moitié de score 1 et l’autre moitié de score 2. Ce taux est concordant avec celui de la littérature (entre 18 et 53,4%) [12-19]. Cette expression était associée à un mauvais pronostic puisqu’elle diminuait significativement la SSR (p=0,001) et qu’elle était corrélée à des facteurs de mauvais pronostic comme la taille tumorale >20mm (p=0,015), le grade III (p=0,002), la négativité des RE (p=0,001), le statut HER2 amplifié (p=0,007), les profils tumoraux «luminal A» (p=0,001), «HER2» (p=0,019) et «triple négatif» (p=0,008), le Ki67≥14% (p=0 ,001), les métastases ganglionnaires (p=0,009), la présence de métastases systémiques au moment du diagnostic (p=0,03), la présence de récidives locales (p=0,001) et systémiques (p=0,004).

Notre étude présente des limites. En effet, il s’agit d’une étude rétrospective qui s’est basée sur des données macroscopiques et histologiques déjà prêtes et utilisées. Toutefois, dans notre analyse, nous avons testé l’immuno-marquage de CD10 de manière «prospective» sur les micro-biopsies et sur des blocs représentatifs des tumeurs. Les résultats de l’expression de CD10 se sont basés sur une classification semi-quantitative, évaluant de manière visuelle la densité des cellules stromales marquées pour classer les carcinomes dans les trois catégories. Cette méthode est subjective et manque de précision mais nous nous sommes basés sur les séries de la littérature qui adoptent la même méthode visuelle d’évaluation. Il n’a pas été rapporté de méthodes de comptage numérique. Les résultats de notre étude sont concordants avec ceux de la littérature. En effet, en ce qui concerne la SSR, les études de Iwaya et Sadaka avaient montré que l’expression stromale de CD10 diminuait significativement la SSR (respectivement p=0,0008 et p<0,001) [13, 14]. Selon Makretsov et Vo, cette expression était un facteur indépendant pour la SSR (p=0,0059, <0,001, <0,01 et 0,003, respectivement) [15, 16].

En ce qui concerne la SG et en analyse univariée, l’étude de Ziadi avait montré que le marquage stromal de CD10 avait tendance à diminuer la SG, sans différence significative (p=0,8), comme c’était le cas dans notre série [17]. En revanche, les études de Iwaya, Makretsov, Sadaka, Kim et Rizk avaient montré que le marquage stromal de CD10 diminuait significativement la SG (p=0,002; p<0,01; p<0,001; p=0,04 et p=0,016) [13-15, 18, 19]. En ce qui concerne l’étude de corrélation entre l’expression stromale de CD10 et les facteurs clinico-pathologiques, six études avaient montré une corrélation significative entre le marquage stromal de CD10 et la taille tumorale >20mm, comme dans notre étude (p=0,015) [12, 16, 18, 20-22]. Plusieurs séries avaient montré une association statistiquement significative entre l’expression stromale de CD10 et le haut grade tumoral (Grade III) comme dans notre série (p=0,002) [3, 12, 13, 15, 17, 18, 21, 22]. Les études de Iwaya, Puri et Vo, avaient en revanche montré l’absence d’une association entre le grade tumoral et l’expression de CD10 [11, 14, 16]. En ce qui concerne les récepteurs hormonaux, plusieurs études avaient montré que l’expression stromale de CD10 était corrélée à la négativité de RE, comme dans notre série (p=0,001) [13-16, 18, 19, 23, 24]. Certaines études avaient montré la présence d’une corrélation significative entre l’expression de CD10 et la négativité des RP [12, 13, 16, 19]. Ce résultat est discordant avec ceux de Makretsov et Ziadi et celui de notre série (p=0,3) [15, 17]. Plusieurs études avaient montré la présence d’une corrélation significative entre l’expression de CD10 et le statut HER2 amplifié, comme c’était le cas dans notre série (p=0,019) [13, 21, 25-27]. En revanche, Makretsov, Rizk, Ziadi et Mohammadizadeh n’avaient pas trouvé de corrélation entre ces deux paramètres [12, 15, 17, 19].

En ce qui concerne le taux de KI67, deux études seulement ont étudié ce paramètre et ont trouvé qu’un taux de Ki67≥14% était significativement associé au marquage stromal de CD10, comme dans notre série (p=0,001) [11, 16]. Deux études ont étudié la corrélation entre le marquage stromal de CD10 et les profils phénotypiques du carcinome mammaire [19, 24]. Dans notre étude, une corrélation significative a été rapportée avec le profil «triple négatif», concordant avec la série de Rizk (p=0,009) et avec les profils «HER2» et «luminal A», concordant avec la série de Jana (p=0,019 et 0,001) [19, 24]. Plusieurs études ont montré que l’expression stromale de CD10 était significativement plus fréquente chez les patientes présentant des métastases ganglionnaires, comme dans notre étude (p=0,009) [3, 12-14, 16-18, 20, 22, 23]. L’étude de Kim avait montré que la positivité de CD10 dans les cellules stromales était plus fréquente chez les patientes présentant une récidive systémique, comme dans notre série (p=0,004) [18]. En ce qui concerne la présence d’emboles vasculaires, Sadaka et Ali ont trouvé que ce paramètre était significativement associé à l’expression stromale de CD10, contrairement à nos résultats [13, 20]. Les données de la littérature ainsi que les résultats de notre étude confirment que dans les carcinomes mammaires invasifs, il existe une association significative entre l’expression stromale de CD10 et les facteurs anatomo-cliniques de mauvais pronostic ainsi que le potentiel évolutif agressif, essentiellement en rapport avec la SSR. Ces résultats sont résumés dans le Tableau 3.

**Tableau 3:** principaux résultats des paramètres significativement corrélés à l’expression stromale de CD10 dans le cancer du sein

Auteurs	Population à l’étude (Carcinomes invasifs/cas contrôles)	Paramètres significativement corrélés au CD10+
**Iwaya et al. 2002**[14]	110 cas/ 13 CIS	Grade III et RE-
**Makretsov et al. 2007**[15]	438 cas /15 CIS	Grade III et RE- SG et SSR diminuées
**Kim et al. 2010**[18]	104 cas/10 CIS	La taille tumorale, Grade III, N+, M+, RE- et SG diminuée.
**Ziadi et al. 2011**[17]	112 cas	N+, Grade III et taille tumorale
**Puri et al. 2011**[11]	50 cas/4 carcinomes mucineux et 1 CIS	HER2 amplifié, ki67 élevé, RE-, RP- et Grade III.
**Mohammadizadeh et al. 2012**[12]	49 cas	RE-, RP-, Taille tumorale, N+ Grade III
**Vo et al. 2013**[16]	73 cas	N+, RE-, RP-, HER2 amplifié, KI67 élevé, taille tumorale et récidives locales
**Jana et al. 2014**[23]	70 cas	Grade III, RE+, HER2 amplifié, Nb mitoses élevé, corrélation inverse au profil “luminal A”
**Taghizadeh-Kermani et al. 2014[21]**	100 cas /50 adénofibromes	Grade III, taille tumorale, N+ et RE-
**Devi BA et al. 2016[22]**	59 cas	Taille tumorale, N+ et Grade III
**Sadaka et al. 2016[13]**	97 cas	RE-, RP-, HER2 amplifié, Grade III, emboles vasculaires + et SSR diminuée
**Ali et al. 2016[20]**	83 cas/ 8 autres types de carcinomes	Grade III, N+, emboles vasculaires+
**Rizk et al. 2017[19]**	60 cas	RE-, RP-, Profil “triple négatif ”
**Louhichi et al. 2018[3]**	133 cas	N+ et Grade III
**Notre série**	57 cas/ 15 CIS	Taille tumorale > 20 mm, Grade III, RE-, Profils luminaux A et B, HER2, et triple négatif. Ki67≥14%, HER2 amplifié, M+, N+, récidives locales et systémiques

**N+ :** métastase ganglionnaire. **M+ :** métastase à distance. **RE-** : Récepteurs aux œstrogènes négatifs. **RP- :** Récepteurs à la progestérone négatifs. **SSR:** Survie sans récidives. **SG :** Survie globale. **CIS:** carcinomes in situ

L’effet pronostique de CD10 serait en rapport avec ses propriétés enzymatiques. En effet, il joue le rôle d’une métallo-endo-peptidase (MEP) ou métallo-protéinase zinc-dépendante qui clive un certain nombre de bio-peptides. Les substrats moléculaires activés ou inhibés contribuent à la régulation de différentes cellules et plus particulièrement les cellules souches [25]. CD10 est utilisé comme marqueur de surface cellulaire pour les cellules souches au niveau de plusieurs tissus normaux (os, moelle, poumon et sein) [25]. Il métabolise des peptides biologiquement actifs tels que la bradykinine, l’ocytocine, le facteur natriurétique auriculaire, la substance P, la bombésine, l’endothéline-1 et la neurotensine [20]. Ces multiples propriétés incriminent le CD10 dans la régulation de nombreux mécanismes physiologiques comme les réactions inflammatoires, l’homéostasie circulatoire et le système neural [27, 28]. Il est capable d’interagir avec des voies de signalisation cellulaires majeures comme la prolifération, la migration et l’apoptose. Au niveau des cellules épithéliales, la méthylation de CD10 induit une augmentation de la migration, de la croissance et de la survie cellulaire [29].

Notre travail présente au moins trois implications. La première concerne la nécessité d’un consensus pour d’étude de l’expression de CD10 dans les cancers du sein, illustrant de manière précise les différentes étapes à suivre et les seuils à considérer. Grâce à ce consensus, l’expression de CD10 pourrait intégrer les comptes rendus standardisés des cancers du sein et les classifications pronostiques. La deuxième implication concerne le volet thérapeutique. En effet, plusieurs études récentes utilisent le micro-environnement tumoral, comme cible thérapeutique avec l’avantage que les cellules du stroma ne sont pas génétiquement instables comme les cellules carcinomateuses et il y aurait ainsi moins de risque de développer des résistances thérapeutiques.

## Conclusion

L’expression de CD10 dans le stroma des carcinomes mammaires invasifs constitue un facteur de mauvais pronostic, prédictif d’une faible survie sans récidives et est associée à un potentiel invasif et métastatique élevé. De nouvelles études multicentriques et prospectives sont nécessaires pour élucider les mécanismes de signalisation, conduisant à une surexpression de CD10 dans le stroma du carcinome invasif du sein. Ces études devraient également aider au développement de thérapies ciblant le CD10 et introduire ce paramètre comme facteur prédictif de réponse à ces traitements. Il serait intéressant d’étudier la variabilité inter-observateur pour l’évaluation de l’expression de CD10 et de développer des méthodes numériques de comptage pour optimiser l’objectivité des résultats trouvés.

### Etat des connaissances sur le sujet

- L’étude de nouveaux marqueurs prédictifs du potentiel invasif et métastatique des cancers du sein est primordiale du fait de son pronostic qui demeure encore grave malgré les progrès diagnostiques et thérapeutiques;
- L’expression stromale de CD10, molécule de la matrice extra-cellulaire serait corrélée à un potentiel agressif de plusieurs types de cancers comme le cancer colorectal, pulmonaire, de la prostate, du rein et du foie, en favorisant la croissance tumorale et l’apparition de métastases;
- Pour les carcinomes mammaires, la valeur pronostique de l’expression stromale de CD10 reste encore controversée avec des résultats discordants.

### Contribution de notre étude à la connaissance

- L’expression stromale de CD10 diminue significativement la survie sans récidives (p=0,001), mais n’a pas d’influence sur la survie globale (p=0,84);
- L’expression stromale de CD10 est significativement corrélée plusieurs facteurs de mauvais pronostique dans le cancer du sein (la taille tumorale >20mm, grade de Scarff Bloom et Richardson III, négativité des récepteurs aux œstrogènes, au statut HER2 amplifié, aux profils phénotypiques «HER2», «triple négatif» et «luminal A», à l’index Ki67≥14%, aux métastases ganglionnaires, à la présence de métastases systémiques au moment du diagnostic et à la présence de récidives locales et systémiques.
